# Investigating Potential GLP-1 Receptor Agonists in Cyclopeptides from *Pseudostellaria heterophylla*, *Linum usitatissimum*, and *Drymaria diandra*, and Peptides Derived from Heterophyllin B for the Treatment of Type 2 Diabetes: An In Silico Study

**DOI:** 10.3390/metabo12060549

**Published:** 2022-06-15

**Authors:** Hui-Jun Liao, Jason T. C. Tzen

**Affiliations:** Graduate Institute of Biotechnology, National Chung Hsing University, Taichung 402, Taiwan; tctzen@dragon.nchu.edu.tw

**Keywords:** *Pseudostellaria heterophylla*, *Linum usitatissimum*, *Drymaria diandra*, cyclic peptides, diabetes, GLP-1, DPP4, Heterophyllin B, Cyclolinopeptide, Diandrine C

## Abstract

GLP-1 receptor agonists stimulate GLP-1R to promote insulin secretion, whereas DPP4 inhibitors slow GLP-1 degradation. Both approaches are incretin-based therapies for T2D. In addition to GLP-1 analogs, small nonpeptide GLP-1RAs such as LY3502970, TT-OAD2, and PF-06882961 have been considered as possible therapeutic alternatives. *Pseudostellaria heterophylla*, *Linum usitatissimum*, and *Drymaria diandra* are plants rich in cyclopeptides with hypoglycemic effects. Our previous study demonstrated the potential of their cyclopeptides for DPP4 inhibition. Reports of cyclic setmelanotide as an MC4R (GPCR) agonist and cyclic α-conotoxin chimeras as GLP-1RAs led to docking studies of these cyclopeptides with GLP-1R. Heterophyllin B, Pseudostellarin B, Cyclolinopeptide B, Cyclolinopeptide C, Drymarin A, and Diandrine C are abundant in these plants, with binding affinities of −9.5, −10.4, −10.3, −10.6, −11.2, and −11.9 kcal/mol, respectively. The configuration they demonstrated established multiple hydrogen bonds with the transmembrane region of GLP-1R. DdC:(cyclo)-GGPYWP showed the most promising docking score. The results suggest that, in addition to DPP4, GLP-1R may be a hypoglycemic target of these cyclopeptides. This may bring about more discussion of plant cyclopeptides as GLP-1RAs. Moreover, peptides derived from the HB precursor (IFGGLPPP), including IFGGWPPP, IFPGWPPP, IFGGYWPPP, and IFGYGWPPPP, exhibited diverse interactions with GLP-1R and displayed backbones available for further research.

## 1. Introduction

### 1.1. GLP-1, GLP-1R Agonists, and Type 2 Diabetes Treatment

Due to urbanization and changes in eating habits, the incidence of type 2 diabetes (T2D) has exploded. As a result, many people’s lives are greatly affected by diabetes, and the complications it causes can further harm health [1]. GLP-1 receptor agonists (GLP-1RAs) and DPP4 inhibitors are incretin-based therapies for type 2 diabetes and are critical second-line drugs for the treatment of T2D. Glucagon-like peptide 1 (GLP-1) and glucose-dependent insulinotropic polypeptide (GIP) are two important incretin hormones that help maintain blood glucose homeostasis [2,3]. Of the two, GLP-1 has received more attention than GIP. GLP-1 is produced and secreted by intestinal enteroendocrine L-cells upon food consumption, and acts on pancreatic islets to stimulate insulin synthesis by activating the GLP-1 receptor (GLP-1R). GLP-1 (7–37) and GLP-1 (7–36) NH2 are their two bioactive forms. Both are rapidly degraded by Dipeptidyl peptidase 4 (DPP4) into GLP-1 (9–37) and GLP-1 (9–36) NH2 after release (t 1/2~1–2 min). The DPP4-degraded form of GLP-1 has relatively low binding affinity with the GLP-1R [2,3]. Studies have observed that patients with type 2 diabetes may not be able to maintain glucose homeostasis due to reduced GLP-1 secretion or accelerated GLP-1 metabolism [4]. Therefore, DPP4 inhibitors have been developed to prolong the effect of GLP-1, or to apply GLP-1 analogs such as exenatide and liraglutide to increase the effect on GLP-1R [5]. According to a meta-analysis, DPP4 inhibitors and GLP-1R agonists affect glucose-lowering and weight control, but GLP-1R agonists are more effective than DPP4 inhibitors [6].

Human GLP-1R is a G-protein-coupled receptor (GPCR) composed of a hydrophilic extracellular domain (ECD) and seven α-helical transmembrane domains (TMD) with 463 amino acids [7]. It is widely expressed in the lung, kidney, heart, pancreatic islets, intestines, multiple regions of the CNS, etc., and is particularly abundant in the pancreatic β-cells. The GLP-1R activation on β-cells generates a series of downstream signal amplification, including rapidly increasing cAMP levels, extracellular Ca^2+^ influx, β-arrestin recruitment, and ERK1/2 cascade. The cAMP-dependent activation of the PKA leads to increased insulin synthesis and release [3,8]. GLP-1R activation in the central hypothalamus has been shown to promote satiety, thereby reducing food intake and slowing gastric emptying [9]. The higher dose of liraglutide (3 mg) under the trade name Saxenda^®^ has been approved to treat overweight and weight-related diseases [10,11]. Semaglutide is being evaluated as a weight-loss drug for obese subjects without diabetes [11]. GLP-1R agonists also have been observed to improve endothelial dysfunction that may occur in diabetic patients through blood-flow-mediated vasodilation [12]. Moreover, studies have demonstrated that GLP-1R agonists can reduce the risk of proteinuria and kidney disease progression [13]. The GLP-1R agonist is therefore considered to bring new prospects for the treatment of T2D and the prevention of its chronic complications [3,8].

**Figure 1:**
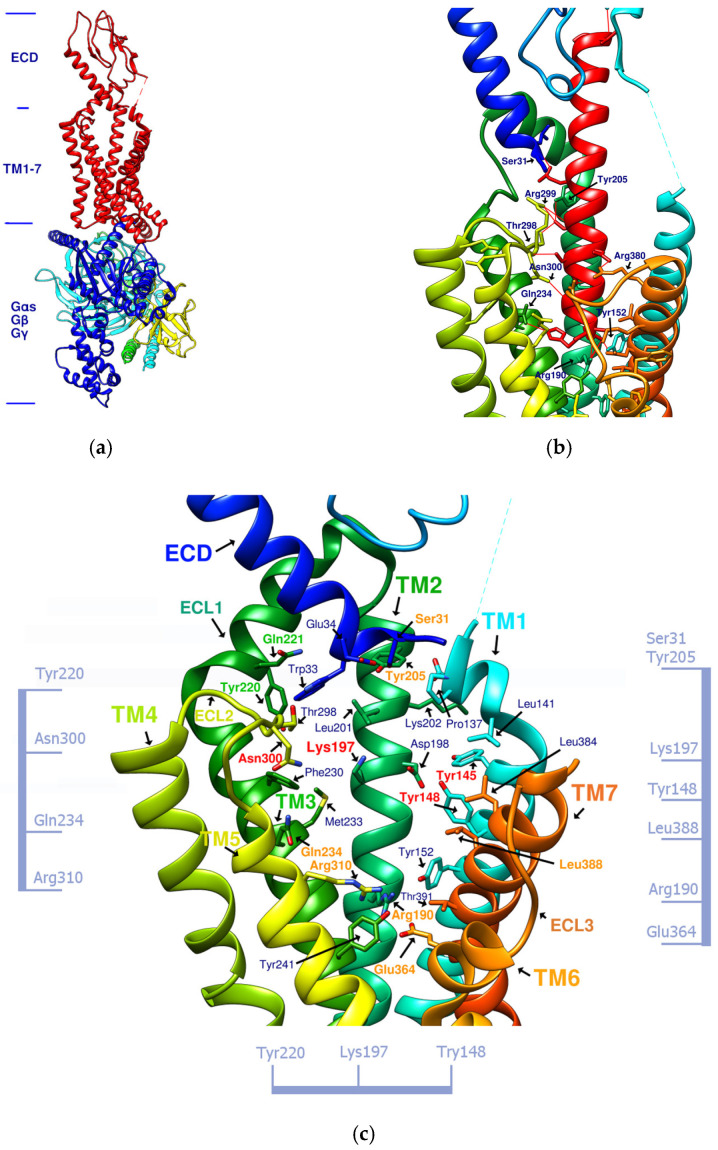
(**a**) GLP-1R and the G-protein (PDB:6X19). (**b**) GLP-1 (red alpha helix, the original ligand of GLP-1R)/GLP-1R (transmembrane region) complex (PDB:6X18). (**c**) Important amino acid residues in the transmembrane region (TM1-7) of GLP-1R (PDB:6X19). Ser31, Tyr205, Gln234, Arg310, Arg190, Glu364, and Leu388, shown in orange, delineate the approximate boundaries of the active site. Asn300, Lys197, Tyr148, and Tyr145, shown in red, are the residues involved in the important hydrogen bonds and π–π interaction network in the middle section. Tyr220 and Gln221, shown in green, are located on ECL1. The labeling of these important amino acids is based on the related studies of GLP-1 agonists [7,21]. The amino acids marked on the vertical axis on both sides and the horizontal axis on the bottom can be used as reference axes for analyzing the configuration of GLP-1R agonists. TM2 can also serve as a reference axis for configurational analysis of GLP-1RAs.

Exendin-4 is a short peptide with 39 amino acids isolated from the venom of the Gila monster. It has a 53% sequence identity with human endogenous GLP-1 and was found to have an agonistic effect on GLP-1R. Exenatide (Byetta^®^, Bydureon^®^) is a synthetic version of exendin-4 and the first hypoglycemic drug classified as a GLP-1 analog [14]. Liraglutide (Victoza^®^) has a 97% homology with human GLP-1. In design, liraglutide conjugates fatty acids to the original GLP-1 structure. This approach strengthens its binding to serum albumin, thereby avoiding rapid degradation by DPP4 and reducing renal clearance for prolonged action. Novel semaglutide (Ozempic^®^) incorporates an absorption enhancer, further realizing the possibility of oral administration [8,15]. The crystal structure of the complex of exendin-4 and GLP-1R shows that except for a part of the C-terminus, exendin-4 still adopts an α-helical conformation, and its binding site is similar to that of GLP-1 [16]. When endogenous GLP-1 penetrates the core of the receptor (PDB:6X18), it interacts with Tyr205, Arg299, Thr298, Asn300, Gln234, Try152, Arg190, etc., resulting in the outward movement of TM6, ECL3, and TM7, and the inward movement of TM1 as well as the reorganization of ECL2, as shown in Figure 1. These displacements later cause the activation of GLP-1R and trigger cAMP production [3,17].

Figure 1b shows that α-helical GLP-1 is inserted vertically from the ECD to TM1-7 in the transmembrane region. From this perspective, all amino acid residues where GLP-1 interacts with GLP-1R can be seen. This perspective best expresses the active site of GLP-1R and is the perspective adopted by most of the relevant literature. Figure 1c lists amino acids on GLP-1R that affect cAMP accumulation or binding affinity. According to studies by K Coopman, D Wootten, and D Yang [18,19,20], essential amino acid residues that affect the binding affinity of GLP-1 or exendin-4 include Y148, Y152, R190, K197, D198, L201, M204, Y205, Q234, Y235, W284, E294, W297, T298, R299, N300, Y305, R310, E364, R380, L384, L388, etc. Residue mutations that may affect cAMP accumulation include Y152, R190, K197, D198, Q234, Y205, W297, N300, R310, E364, T391, etc. These amino acid residues are more numerous than those that interact with GLP-1, and their interactions are also considered when developing new GLP-1R agonists.

### 1.2. Development of Small-Molecule Nonpeptide GLP-1R Agonists

**Figure 2:**
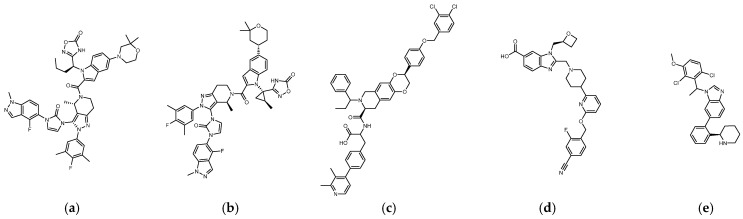
(**a**) UK1 (PDB:6X19; MW: 886 g/mol), (**b**) LY3502970 or V6G, OWL-833 (PDB:6XOX; MW: 883.0 g/mol), (**c**) TT-OAD2 (PDB:6ORV; MW: 929.7 g/mol), (**d**) PF-06882961 or UK4 (PDB:6X1A; MW: 555.6 g/mol), (**e**) LSN3160440 or QW7 (PDB:6VCB; MW: 480.43 g/mol).

**Figure 3:**
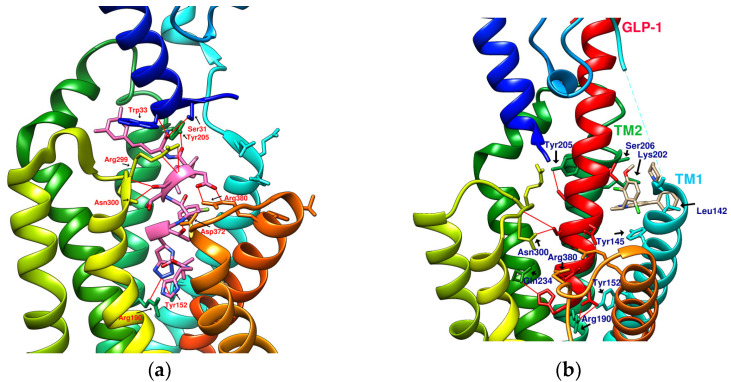

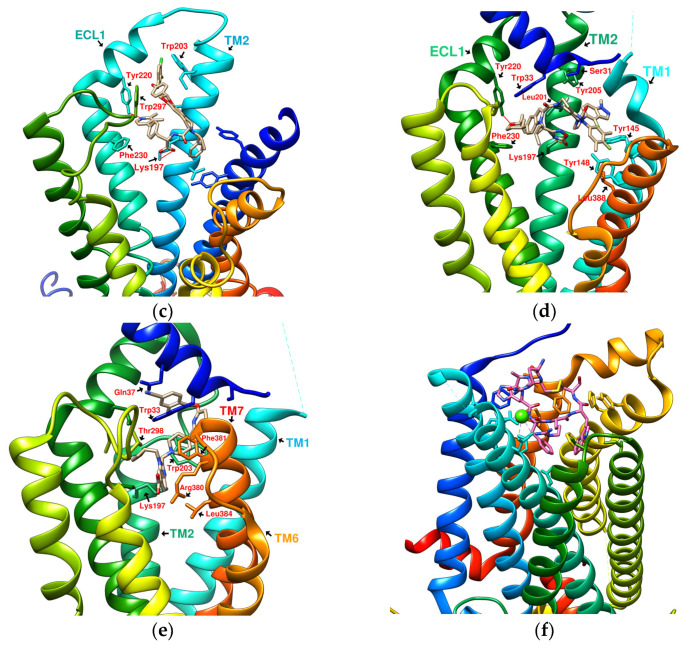
Reference GLP-1R and MC4 (GPCR) complex (PDB file from RCSB Protein Data Bank). (**a**) Peptide 5/GLP-1R (PDB:5NX2); (**b**) LSN3160440 and GLP-1 in GLP-1R (PDB:6VCB); (**c**) TT-OAD2/GLP-1R (PDB:6ORV); (**d**) LY3502970 in GLP-1R (PDB:6XOX); (**e**) PF-06882961/GLP-1R (PDB:6X1A); (**f**) Setmelanotide/MC4R (PDB:7PIU).

The reported GLP-1R agonists include early polypeptide agonists with a secondary structure, truncated free short peptides, and novel nonpeptide small molecules (Figure 2). Although semaglutide has dosage forms for oral administration, the development of small oral nonpeptide GLP-1RA has always been one of the prevalent issues for new drug research and innovation in addition to GLP-1 analogs. According to A Jazayeri et al., it was found that peptide 5 (PDB:5NX2) modified from the GLP-1 truncated sequence (8-17) can produce total agonist activity on GLP-1R [7]. Peptide 5 establishes a hydrogen bond with Ser31, Asn300, Tyr152, Arg190, etc., on GLP-1R, which is similar to the partial interaction between GLP-1 and GLP-1R. The crystal of peptide 5 appears to be a linear peptide and no longer has an α-helical structure. Its top end is fixed with a cap that passes through Trp33 and turns to ECL1 instead of extending to ECD as GLP-1 does (Figure 3) [7]. The GLP-1R–GLP-1–LSN3160440 complex (PDB:6VCB) reveals a novel drug design concept for the activation of GLP-1R [22]. LSN3160440 is an allosteric modulator with molecular glue or uncompetitive pharmacology, which only occupies the local interval between TM1 and TM2 and needs to cooperate with GLP-1 (9–36) NH2 to produce enough effect. At a concentration of 1 μM, it can convert GLP-1 (9–36) NH2 from a 3% effective partial agonist to a full agonist [22]. Recently, nonpeptide GLP-1R agonist PF-06882961, also known as UK4 (PDB:6X1A); LY3502970, also known as OWL-833, V6G (PDB:6XOX and PDB:7E14); and TT-OAD2 (PDB:6ORV) were found entirely out of a GLP-1 peptidomimetic basis. UK1 (PDB:6X19) and V6G (PDB:6XOX) are the ligands of GLP-1R with similar structures [17,21,23,24]. The molecular weights of LY3502970, TT-OAD2, and PF-06882961 are 883.0, 929.7, and 555.6 g/mol, respectively. TT-OAD2 and PF-06882961 have etheric bonds in their structures, which increase the flexibility of the molecule. LY3502970 and UK1 have multiple side chains on the backbone. There are multiple hydrogen bond donors and acceptors in their structures, which are beneficial for interacting with GLP-1R. These molecules bind to the middle-upper regions of the GLP-1R transmembrane helix rather than extending straight to the lower area around Arg190 (Figure 3).

TT-OAD2 adopts a U-shaped orientation on GLP-1R. Its backbone extends from Lys197 to Tyr220, sitting between TM2, TM3, and ECL1 [24]. LY3502970 (OWL-833/V6G) [21] displays a three-branch conformation to interact with residues on ECD, TM1, TM2, TM3, and ECL2 (similar to the role of UK1 in PDB:6X19); thus, the range of its influence is greater than that of TT-OAD2. According to T Kawai et al., LY3502970 reacts as a more potent cAMP agonist than TT-OAD2 [21]. The configuration of PF-06882961 in GLP-1R (PDB:6X1A) shows that one end occupies the vicinity of TM7, and the other end reaches ECL1 after passing Try33 of the ECD. LY3502970 shows more binding affinity with GLP-1R than PF-06882961. However, in the study, the concentration-response curves of these two agonists on cAMP accumulation were similar (Figure 3) [17]. These small-molecule agonists may impact Lys197, Tyr145, and Tyr148 in the central area of GLP-1R or establish a connection with the ECL1 region, in which case they need to pass through Trp33 and Thr298. Lys197 is an important residue for these agonists to form hydrogen bonds in the center, and Tyr145, Tyr148, and Trp33 are the primary targets for constructing π–π interactions. These three molecules use different strategies to achieve the activation of GLP-1R, suggesting that the activation of GLP-1R has a certain degree of flexibility. Regarding the space, conformation/configuration, and interaction of these molecules with GLP-1R, there may be more possibilities to design new GLP-1R agonists. 

In addition to activating cAMP, PF-06882961 has also been shown to promote β-arrestin recruitment, ERK1/2 phosphorylation, and calcium mobilization. Compared with PF-06882961, these responses in LY3502970 are lacking or extremely weak, and related studies have explained that the difference may be due to more interactions between PF-06882961 and TM7 [17]. β-arrestin plays a role in GPCR desensitization (loss of response caused by prolonged use of agonists). β-Arrestin 1-mediated ERK1/2 activation may also be beneficial in protecting β-cells from apoptosis [3,25]. Since the axes of TM5, TM6, and TM7 on GLP-1R are skewed outwards, small molecules that appear in the upper part of the active site have difficulty interacting with amino acids on these three axes. When one end of the molecule tilts to the middle and down to the vicinity of Leu388 and Thr391, the possibility of interacting with TM5-7 will increase, and the corresponding biased activation may be manifested accordingly, as shown by PF-06882961 (Figure 3e). 

### 1.3. Reference Case of Cyclic Peptides (Cyclopeptides) as GPCR Agonists/Antagonists

Joakim E. Swedberg et al. found that monocyclic or bicyclic α-conotoxin peptidomimetic chimeras act as potent agonists of GLP-1R [26]. They found effective interactions between cyclopeptides and GLP-1R. However, there is currently no protein crystal structure for this study, and there is no way to see the actual configuration of these cyclopeptides on GLP-1R. Melanocortin 4 receptor (MC4R) provides another case study for exploring GPCR agonists/antagonists. MC4R belongs to class A GPCRs, is located in the hypothalamus area, and plays a vital role in appetite and energy control. The defect of MC4R has been observed to cause a lack of satiety and early onset of severe obesity syndrome. Setmelanotide (Ac-Arg-Cys(1)-D-Ala-His-D-Phe-Arg-Trp-Cys(1)-NH2, MW: 1,1117.3 g/mol) is a highly potent MC4R agonist. The endogenous ligand for MC4R is the tridecapeptide “α-melanocyte-stimulating hormone”, or α-MSH for short. The structure of setmelanotide includes the α-MSH tetrapeptide pharmacophore (His6-Phe7-Arg8-Trp9) and four other amino acids to form an octacyclic peptide containing a pair of disulfide bonds. Compared with α-MSH, setmelanotide can effectively activate the defective MC4R, which has been proven to help with weight control of the MC4R-variant carrier [27]. SHU9119 is an MC4R antagonist. Its molecular formula “Ac-Nle(4)-c(Asp(5)-2′-Nal(7)-Lys(10))α-MSH(4-10)-NH2” also contains truncated α-MSH. PDB:6W25 and PDB:7PIU show the structures of MC4R in complex with SHU9119 and setmelanotide (Ca^2+^ as a cofactor), respectively, demonstrating the ability of cyclic peptides (cyclopeptides) to interact well with multiple α-helices of GPCRs (Figure 3f) [28,29]. On the other hand, peptide 5 (PDB:5NX2) consists of a truncated sequence (8–17) from GLP-1 that makes it a GLP-1R agonist, but did not undergo a cyclization process [7].

### 1.4. Correlation between GLP-1R Active Site and Accommodated Molecular Size

The linear, multi-branched, or cyclic configurations displayed by various GPCR agonists illustrate that an agonist needs to interact with multiple amino acid residues to activate a GPCR. Therefore, GLP-1RA generally has a specific molecular size. PF-06882961 with an MW of 555.6 g/mol exhibits the characteristics of a complete GLP-1R agonist, whereas LSN3160440 with an MW of 480.43 g/mol is set as an ectopic modulator. GLP-1 agonists with a molecular weight of less than 480 g/mol may not be easy to design. The region’s union, occupied by various GLP-1RA, can be approximately framed by the following amino acids: Ser31, Tyr220, Asn300, Gln234, Arg190, Glu364, Leu388, Pro137, and Leu141. These amino acids define a hemispheric-like region. The area is large enough to accommodate molecules about the size of a decapeptide, such as peptide 5 (PDB:5NX2), or a cyclic peptide of a specific size. If the circumference of the cyclic peptide is within this range, it may have a chance to enter.

### 1.5. The Research Potential of Pseudostellaria heterophylla, Linum usitatissimum, and Drymaria diandra as Natural Hypoglycemic Products and GLP-1R Agonists

Several studies have shown that *Pseudostellaria heterophylla* (Taizishen or *P. heterophylla*), *Linum usitatissimum* (*L. usitatissimum*, flaxseed, flax, linseed), and *Drymaria diandra* (*D. diandra*, *Drymaria cordata*, *D. cordata*) are three plant products that have the effect of lowering blood sugar [30,31,32,33,34,35]. The three plants have in common that they are rich in cyclopeptides [30,36,37,38,39,40,41]. Their cyclopeptides are called “Caryophyllaceae-type cyclopeptides” (CTCs) in the study by NH Tan et al. [40] or “orbitides” in the survey by PG Arnison et al. [41]. These plant N-C cyclic peptides are mainly monocyclic peptides consisting of hydrophobic amino acids and do not contain disulfide bonds. The main components of *P. heterophylla* include saponins, polysaccharides, and cyclopeptides. Early herbal pharmacopeias state that *P. heterophylla* can be used to improve fatigue, polydipsia, lung diseases, and physical fitness [30,36]. In modern times, it appears in Chinese medicine as compound prescriptions for the treatment of type 2 diabetes or as a nutritional supplement for the weak after an illness. The primary ingredients of *L. usitatissimum* (flaxseed) include alpha-linoleic acid, lignans, and cyclopeptides. Studies have shown that it has antidiabetic, anticancer, antimalaria, cardioprotective, and immunomodulatory properties [37]. *Drymaria diandra* was used to treat diabetes in the Sikkim area of India. In an experiment conducted by S. Patra et al., *Drymaria cordata* (*D. diandra*) methanol extract (DCME) was administered to diabetic rats. It was observed that FBG, HbA1c, serum lipids, and related liver and kidney antioxidant parameters decreased. Compared with the diabetes group, the β-cell density of the pancreatic tissue in the treatment group increased [35].

Targets of hypoglycemia in which natural products are often discussed include PTP1B, α-glucosidase, α-amylase, PPARγ, GLUT4, DPP4, etc. [42]. The volume of cyclopeptides in these three plants is larger than that of flavonoids, polyphenols, and terpenes. The active sites of the DPP4 and GLP-1 receptors, both targets of incretin-based therapy, are large enough to accommodate these plant-derived cyclopeptides [43,44]. The active sites of PTP1B, α-glucosidase, α-amylase, PPARγ, and GLUT4 are small compared to DPP4, and large cyclopeptides are not easily accessible. DPP4 inhibition is a mechanism by which many natural products are involved in promoting hypoglycemic effects. We have previously observed the possible impact of *P. heterophylla*, flaxseed, and *D. diandra* cyclopeptides on DPP4 through molecular modeling. Most of the cyclopeptides in these plants could have binding affinities better than −9.0 kcal/mol to DPP4. The docking configuration demonstrates that these cyclopeptides can approach and interact with important amino acids near the catalytic center [45]. Compared to DPP4, the role of natural products on GLP-1R is much less discussed [42]. It may be because general small-molecule natural substances do not easily establish hydrogen bonds with multiple α-helices at the active sites of GPCRs. Cyclopeptides may not be able to configure properly in a small active site, but in the case of DPP4, GLP-1R, etc., with a large active region, cyclopeptides may be able to exert their structural properties [44]. The cyclopeptides are not easy to separate and purify, and GLP-1R does not have commercially available reagents such as DPP4, which increases the threshold for studying the effect of cyclopeptides on GLP-1R. The advent of protein crystals of small, nonpeptidic GLP-1R agonists has provided insight into how small molecules without α-helical structures act at the GLP-1 receptor. Multiple cyclopeptides from these three plants are similar in molecular size to LY3502970 and TT-OAD2. The centroids provided by the ligands of protein crystals make it possible to analyze the interaction of natural compounds with GLP-1R by molecular modeling. In docking, the location of these cyclopeptides at GLP-1R can be observed and compared to the positions of LY3502970 and TT-OAD2 in protein crystallization.

DPP4′s active site is located outside the cell membrane, and molecules do not need to enter the cell membrane to interact with it [44]. According to research, GLP-1R agonists (GLP-1RAs) have a greater impact on hemoglobin A1c (HbA1c), fasting plasma glucose (FPG), blood lipids, and body weight in patients with type 2 diabetes than DPP4 inhibitors [6]. GLP-1R is the target of incretin-based therapy that merits particular emphasis and study. However, the interaction with GLP-1R involves crossing the cell membrane, which is a more complicated process than the interaction with DPP4. Nonpeptide GLP-1R agonists currently in development interact with amino acid residues in the α-helical transmembrane domain of GLP-1R; therefore, the cell membrane permeability of the compound should be considered when designing new GLP-1RAs. These plant-derived cyclopeptides are nonpolar, mainly composed of hydrophobic amino acids, and have no additional glycosylation or methylation modifications. Commonly, nonpolar cyclopeptides enter cells via passive transport [46,47]. Methylated cyclopeptides may exhibit accelerated passive transport when moving across membranes [48]. Unlike passive transport, cell-penetrating peptides (CPPs) consisting of three to four arginine residues and one to two hydrophobic residues can induce deformation of the cell membrane and then be taken up by cells via endocytosis [49]. Plant cyclopeptides that do not have the conditions to constitute a CPP are unlikely to disrupt cell membranes through endocytosis. Unmethylated nonpolar cyclopeptides may stay in the cell membrane for some time before passing through, and this membrane crossing process may provide an opportunity to facilitate their interaction with GLP-1R [50]. 

### 1.6. Exploring the Possibility of HB Linear Precursor “IFGGLPPP” Derivatives Acting on GLP-1R

The GLP-1 truncated sequence (8~17) of peptide 5 (PDB:5NX2) presents a linear peptide without a secondary structure as an example of a GLP-1R agonist. Heterophyllin B (HB) is the most famous and most studied cyclopeptide in *P. heterophylla*. “IFGGLPPP” is a linear peptide precursor of Heterophyllin B proposed by W Zheng et al. [51]. In our previous docking study of DPP4, the discussion covered HB’s linear precursor IFGGLPPP and a series of its derivatives. Among them, the binding energy of IFGWPPP to DPP4 was as good as −12.0 kcal/mol [45]. We also wanted to know whether peptide derivatives of IFGGLPPP could have an affinity to GLP-1R similar to the linear peptide 5 and whether it could potentially serve as a backbone for a GLP-1R agonist. The principles of designing DPP4 inhibitors and GLP-1R agonists with IFGGLPPP as the backbone are different, as a DPP4 inhibitor blocks the catalytic center and avoids cleavage by DPP4, whereas a GLP-1R agonist requires multiple interactions with the seven helical axes.

Molecular modeling by AutoDock Vina was used in this study to investigate the potential of a series of cyclopeptides from three plants and HB-derived peptides as GLP-1R agonists. The correlation between the test compound and GPCR is far more complicated than the evaluation of binding energy because GPCR activation involves complex issues, such as signaling bias, structure-activity relationship, etc. The prediction of the binding affinity score cannot be entirely or directly equivalent to the possible influence on cAMP activation. Molecular modeling still gives us a chance to explore potential targets for cyclopeptides at the preliminary stage. The results of many cyclopeptides and a series of linear peptides docking with GLP-1R provide a window for observing various possible configurations of peptide compounds in GLP-1R. There will be opportunities for more in-depth research on compounds discovered or screened out through the docking process. 

## 2. Results and Discussion

### 2.1. Prediction of the Binding Affinity of Plant-Derived Cyclopeptides Docking with GLP-1R

A series of cyclopeptide summaries from *P. heterophylla* (HA~PH), flaxseed (CLA~CLF), and *D. diandra* (DmA~DdC), including abbreviations, molecular formulas, and the binding affinity (BA) for docking with GLP-1R, are listed in Table 1. The docking results showed that HB, PB, CLA, CLC, DmA, and DdC had good binding affinity with GLP-1R, with values of −9.5, −10.4, −9.9, −10.6, −11.2, and −11.9 kcal/mol, respectively. These also happen to be the cyclopeptides with higher content, or are more common, in the three plants. It is speculated that the interaction with GLP-1R may be one of the reasons why these plants can regulate blood sugar. With good binding affinity to GLP-1R, they may also have potential as nutritional supplements to assist in weight management. DmA, CLB, and CLC of the nonapeptide and PB of the octapeptide showed good docking affinity with GLP-1R because macrocyclic peptides can have multiple interactions with GLP-1R. Among these plant-derived cyclopeptides, DdC:(cyclo)-GGPYWP-(cyclo) is a hexacyclic peptide with a much smaller structure than DmA and had the highest binding affinity. DdC can obtain good docking affinity, but this cannot be seen from the sequence composition alone; thus, DdC required further analysis of its configuration and interaction with GLP-1R.

### 2.2. The Research Potential of DdC as GLP-1RA and Its Configuration Characteristics in Docking 

**Figure 4:**
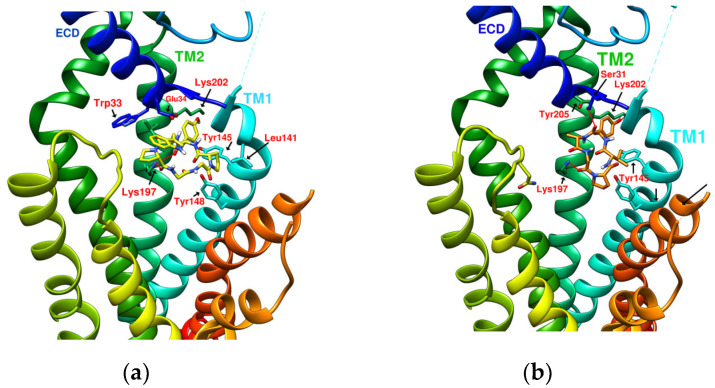
(**a**) DdC/GLP-1R (−11.9 kcal/mol), (**b**) HJ/GLP-1R (−9.7 kcal/mol).

**Figure 5:**
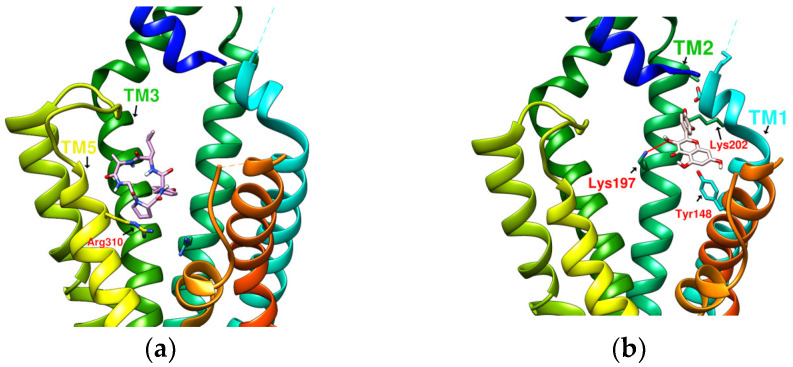
(**a**) PA/GLP-1R (−8.1 kcal/mol), (**b**) Quercetin/GLP-1R (−7.8 kcal/mol).

**Figure 6:**
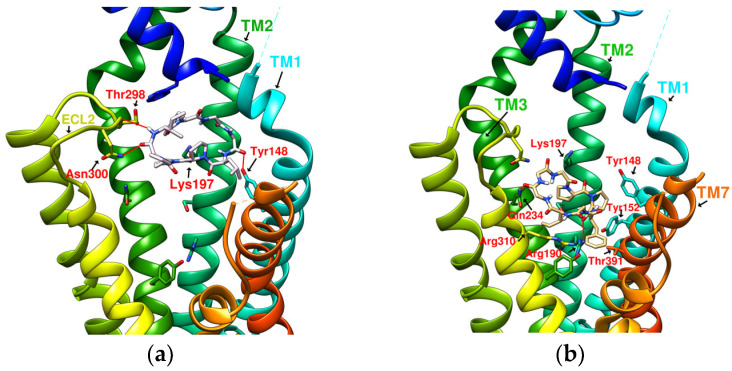
(**a**) HB/GLP-1R (−9.5 kcal/mol), (**b**) PB/GLP-1R (−10.4 kcal/mol).

**Figure 7:**
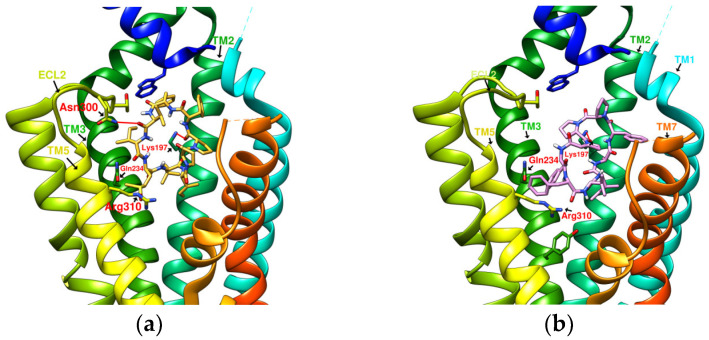
(**a**) CLC/GLP-1R (−10.6 kcal/mol), (**b**) DmA/GLP-1R (−11.2 kcal/mol).

**Figure 8:**
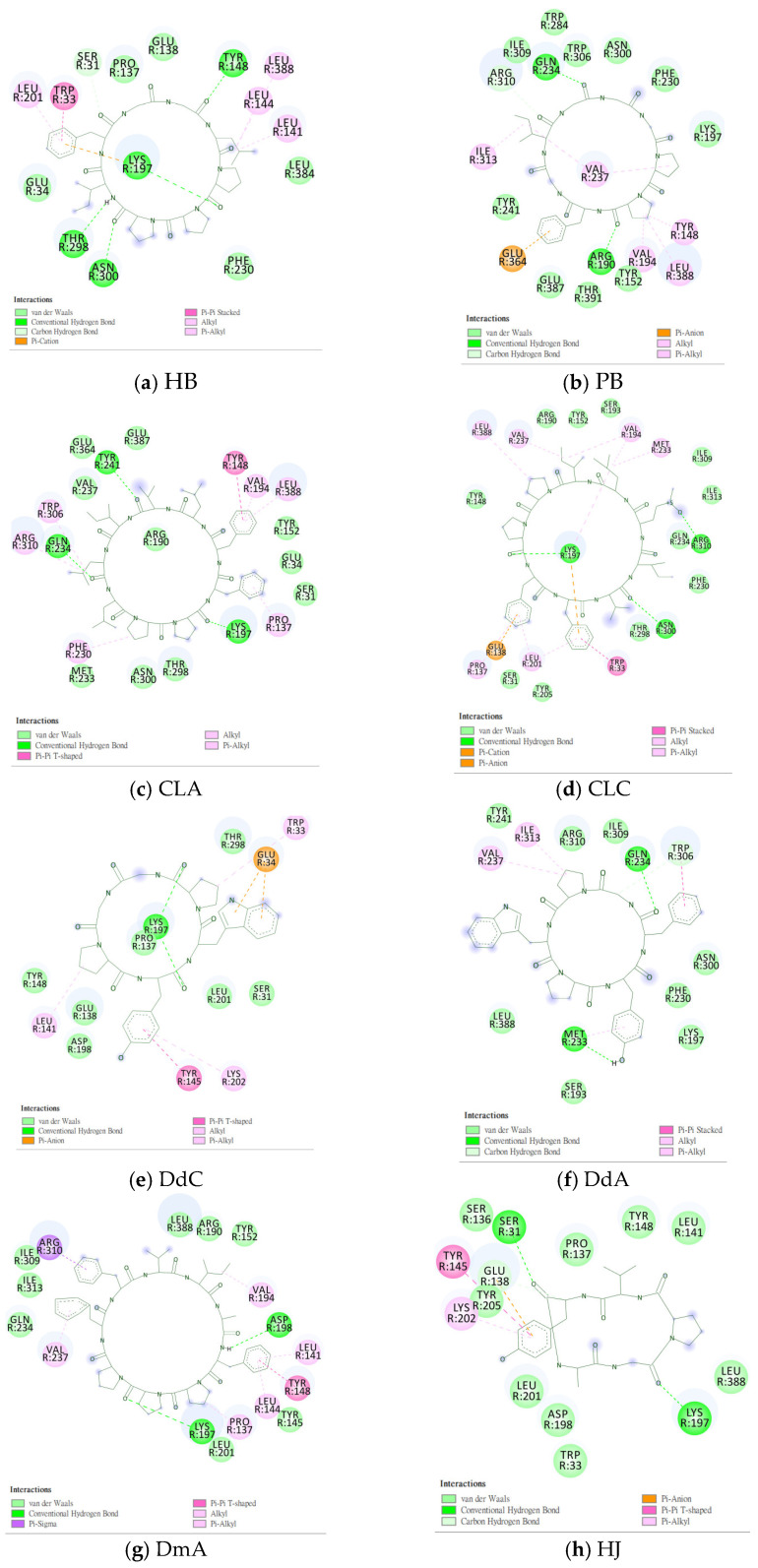
“Receptor–ligand interaction” of cyclopeptides from the three plants with GLP-1R. (**a**) HB; (**b**) PB; (**c**) CLA; (**d**) CLC; (**e**) DdC; (**f**) DdA; (**g**) DmA; (**h**) HJ.

The docking results of the cyclopeptide and GLP-1R are presented in 3D, as shown in Figure 4, Figure 5, Figure 6 and Figure 7 (hydrogen bonds are shown as red lines). Discovery Studio Visualizer was used to demonstrate 2D maps of ligand–receptor interactions, including hydrogen bonding and hydrophobic interactions, shown in Figure 8. The structures of the cyclopeptides can be seen from these 2D plots and correspond to the amino acid sequences in Table 1. Reading the 3D and 2D plots together clarifies which amino acid on the cyclic peptide interacts with which residue of GLP-1R. In Figure 4, Figure 5, Figure 6 and Figure 7, the two graphs are side-by-side because the two graphs are meaningful for comparison. Figure 4a and Figure 8e document the DdC docking configuration and ligand–receptor interaction. In the interaction between DdC and GLP-1R, two hydrogen bonds were displayed between P^(DdC)^, W^(DdC^^)^, and Lys197^(GLP−1R^^)^ (Figure 8a,d,g show that HB, CLC, and DmA each have only one hydrogen bond with Lys197). In addition, Y^(DdC^^)^ and W^(DdC^^)^ established π interactions with Glu34^(GLP−1R)^ and Tyr145^(GLP−1R)^, respectively. DdC has a simplified and valuable hexacyclic peptide structure, in which two glycines and two prolines are helpful to the formation of β-turns, and in the case of establishing a hydrogen bond with Lys197, the angles of Y^(DdC)^ and W^(DdC)^ can simultaneously generate π attraction with the surroundings. Therefore, DdC developed a strong binding affinity with GLP-1R.

### 2.3. The Predicted Configuration of Other Cyclopeptides Besides DdC in the Active Site of GLP-1R 

From the 3D renderings shown in Figure 1, it was found that Tyr205, Lys197, and Arg190 on the TM2 axis can be used as reference coordinates to observe the relative relationship between the docking result and GLP-1R. The main structures (predicted or crystallized) of DdC, TT-OAD2, LY3502970, PF-06882961, and HB were distributed in the area above Lys197 (from the positive perspective of the axis). Compared with the predicted result of DdC, the configuration of HB was shifted to ECL2. In addition to the interaction with Tyr148, Lys197, and Trp33, it also had hydrogen bonds with Asn300 and Thr298. Larger cyclopeptide molecules, such as CLC and DmA, had a configuration that surrounded Lys197 in the center and interacted with Arg310^TM5^ on the periphery. The docking position of PB was in the area below Lys197, which interacted with Gln234^TM3^, Arg190^TM2^, Tyr152^TM1^, and Glu364^TM6^. HB, PB, CLC, DmA, and DdC were the more distinctive or abundant components in these plants. The docking results showed that their molecules can be distributed in the transmembrane cavity of GLP-1 in a position suitable for their conditions, and establish hydrogen bonds or π–π interactions with the surroundings. The possibility of their interaction with GLP-1R may constitute one of the reasons why these natural products have hypoglycemic effects. The layout of PB in GLP-1R is a particular case. Since there was a relatively large space above Lys197, most of the cyclopeptides and LY3502970 appeared in this area. It was previously reported that PF-06882961 had been observed to promote the recruitment of β-arrestin at a physiological level due to its relationship with TM7. According to the analysis conducted using DS Visualizer, none of these series of plant cyclic peptides had hydrogen bonds with the amino acid residues of TM7, but they may have hydrophobic effects. The location of PB allowed it to interact with low-position residues, such as Tyr152^TM1^ or Glu364^TM6^. Tyr152 and Glu364 were adjacent to the TM7 axis; thus, the configuration of PB was more relevant to TM7 in these cyclopeptides.

In Table 2, cyclopeptides are compared with small-molecule nonpeptidic GLP-1R agonists. Interactions of TT-OAD2, LY3502970, and PF-06882961 with GLP-1R were obtained from the literature records [17,21,24], including hydrogen bonding and hydrophobic interactions (hydrogen bonds are marked with red lines in Figure 3). Cyclopeptides have many residues involved in hydrophobic interactions, thus only hydrogen bonds and π interactions were recorded (Figure 8). Cyclopeptides (in Table 2), TT-OAD2, LY3502970 and PF-0688296 showed more interactions with TM1 and TM2. These molecules all interacted with at least the three helical axes of GLP-1R. CLC and DmA can act on TM5 due to their larger radius. PB was located in a lower position to interact with TM6. The three nonpeptide inhibitors interacted with Y220 or Q221 of ECL1, but none of the cyclopeptides (only had hydrogen bonds with ECL2). 

### 2.4. Comparing Configurations of DdC, LSN3160440, Quercetin, HJ, and PA on GLP-1R

In the docking with GLP-1R, the predicted DdC appeared in a relatively limited area compared to the lowest energy configurations of CLC and DmA. Why does DdC:(cyclo)-GGPYWP-(cyclo), obtain the best binding affinity? When the linear peptides “PYWP” and “GGPYWP” were used as ligands for GLP-1R, the docking results indicated that without the constraint of the ring, the original π interaction between DdC and GLP-1R disappeared. Although there were still hydrogen bonds formed, the binding affinity was greatly reduced. The comparison between DdC and its linear sequence suggests that the flexibility of glycine^DdC^ and the rigidity of proline^DdC^ plus the constraint of the ring induce the possibility of a specific conformation to help the stable binding between DdC and the receptor. Moreover, cyclopeptides provide favorable conditions for the formation of π interactions in a confined space, which helps enhance all binding affinity other than the effect of hydrogen bonds. 

The studies by Ana B. Bueno et al. [22] revealed that LSN3160440 (MW: 480.43 g/mol) is an allosteric modulator of GLP-1R, which acts as a protein–protein interaction (PPI) stabilizer or molecular glue to assist in the adhesion of inactive GLP-1 (9-36) NH2 on GLP-1R. LSN3160440 does not have the characteristics of a complete agonist but plays a role in the GLP-1R–LSN3160440–GLP-1 ternary complex. Meanwhile, their research confirmed that the hydrogen bond network between T13^GLP−1^ and Y148^TM1^, D198^TM2^, and K197^TM2^ has a great influence on the activity of GLP-1. In order to coexist with GLP-1, LSN3160440 retreats to the vicinity of Lys202 and Leu142, as shown in PDB:6VCB [22]. The position of DdC (MW: 657.7 g/mol) predicted by docking can be defined by Lys202, Tyr145, Tyr148, Lys197, Trp33, and Glu34 on GLP-1R. Since the molecular weight of DdC was larger than that of LSN360440, its predicted configuration was displayed in the broader region and partially overlapped with the position of GLP-1 in the ternary complex. Therefore, the interaction between DdC and GLP-1R can involve the critical amino acid residue Lys197 in the hydrogen bond network, which is not available in LSN3160440. 

The case of quercetin (MW: 302.24 g/mol) docking with GLP-1R showed that its predicted configuration appeared between Lys202 and Tyr148 and formed hydrogen bonds with Lys197. However, the binding affinity obtained was only −7.8 kcal/mole due to the small size of the molecule. According to an early study by C Koole et al., it was found that quercetin selectively modulates calcium (Ca^2+^) signaling in the presence of truncated GLP-1 but has no effect on cAMP accumulation [52]. The MW of HJ (487.5 g/mol) in Table 1 was close to that of LSN3160440 (480.43 g/mol). The position of HJ when docking was almost the same as the position of DdC. Compared with PA (which has a sequence similar to HJ), the position of HJ seemed to be more conducive to increasing binding affinity. The docking configurations of quercetin, HJ, and DdC showed that the interval between Lys202, Tyr148, and Lys197 was one of the stable positions where some molecules may tend to appear in GLP-1R. In this interval, the ligand has the potential to have π–π interactions with Tyr145, Tyr148, and Trp33 and form hydrogen bonds with Tyr205, Lys197, Ser31, etc. DdC made a good demonstration, showing most of the advantages in this interval. 

### 2.5. Prediction of Binding Affinity of IFGGLPPP Peptide Derivatives to GLP-1R

**Figure 9:**
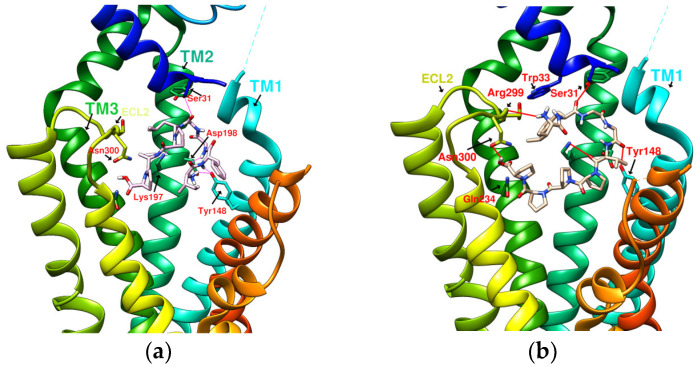
(**a**) IFGGLPPP^N4^ (−8.9 kcal/mol), (**b**) IFGGLPPPP^N5^ (−9.6 kcal/mol).

**Figure 10:**
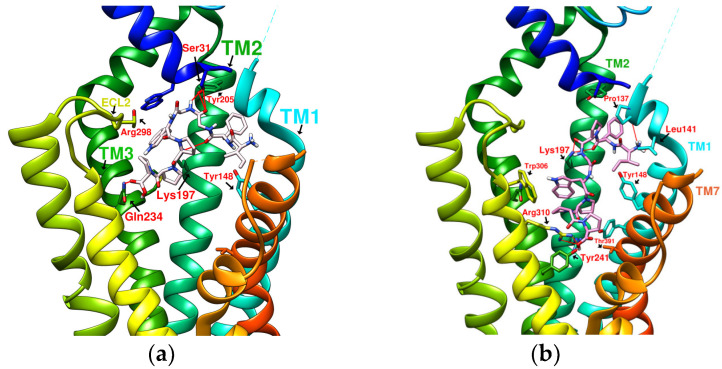
(**a**) IFGGWPPP^N7^ (−10.7 kcal/mol), (**b**) IFPGWPPP^N11^ (−11.0 kcal/mol).

**Figure 11:**
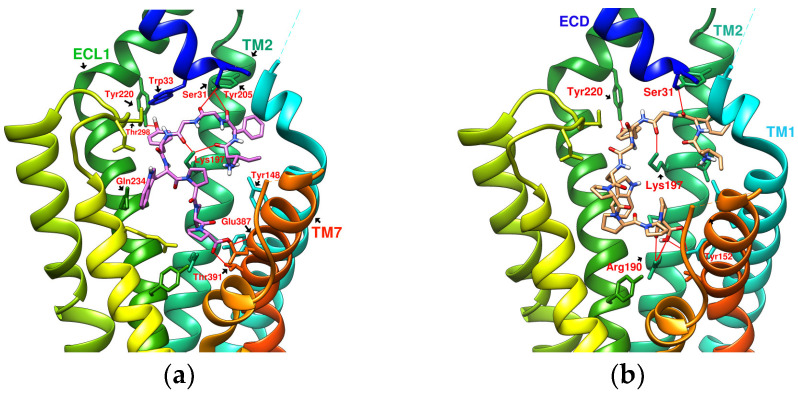
(**a**) IFGGYWPPP^N20^ (−10.9 kcal/mol), (**b**) IFGYGWPPPP^N21^ (−11.7 kcal/mol).

**Figure 12:**
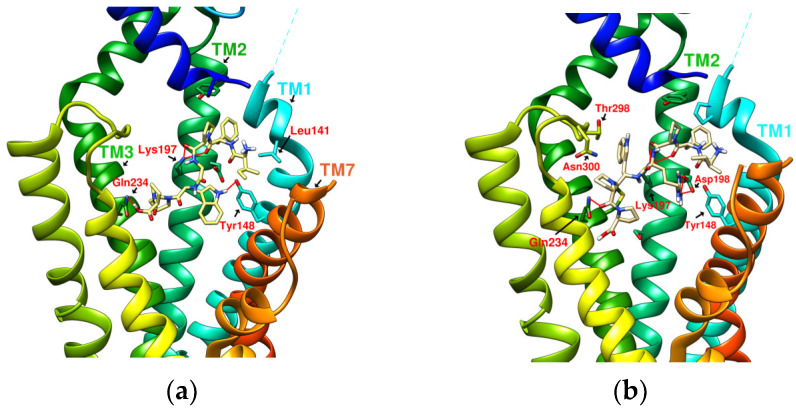
(**a**) IFPGWPP^N10^ (−10.4 kcal/mol), (**b**) IFPRWPP^N13^ (−10.4 kcal/mol).

**Figure 13:**
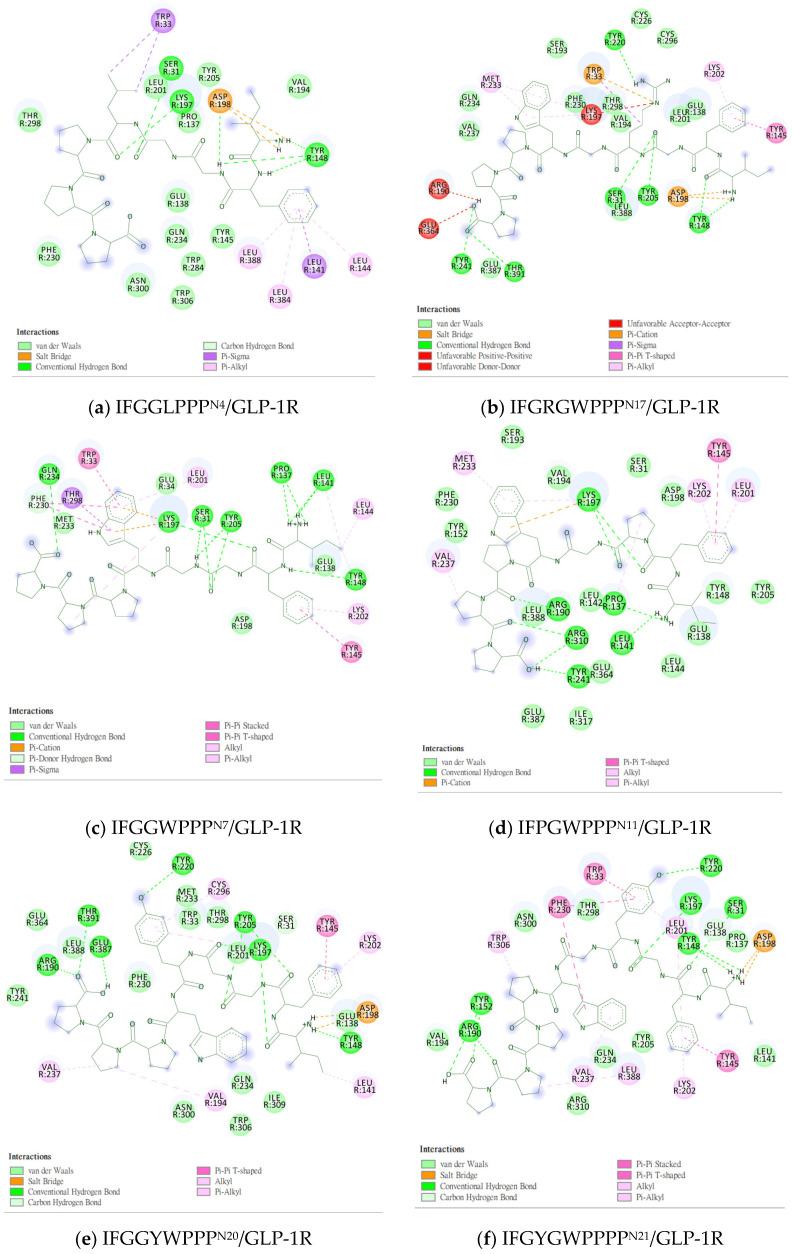
“Receptor–ligand interaction” of IFGGLPPP-derived peptides with GLP-1R from DS Visualizer. (**a**) IFGGLPPP^N4^/GLP-1R; (**b**) IFGRGWPPP^N17^/GLP-1R; (**c**) IFGGWPPP^N7^/GLP-1R; (**d**) IFPGWPPP^N11^/GLP-1R; (**e**) IFGGYWPPP^N20^/GLP-1R; (**f**) IFGYGWPPPP^N21^/GLP-1R.

The test sequence of IFGGLPPP-derived peptides is listed in Table 3 with interaction analysis from DS Visualizer. Figure 9, Figure 10, Figure 11, Figure 12 and Figure 13 show the 3D configurations (H-bonds: red lines) and 2D interaction plots of representative derivatives. IFGGLPPP-derived peptides displayed multiple configurations with good binding affinity when docked to GLP-1R. GGPYWP^(N2)^, AFPPPFFVI^(N3)^, and IFGGLPPP^(N4)^ are the linear forms of DdC, DmA, and HB, respectively. The backbone of IFGGLPPP (linear precursors of HB) was the most promising. When IFGGLPPP was docked to GLP-1R, the binding affinity was −8.9 kcal/mol. Receptor–ligand interaction analysis revealed hydrogen bonding between IFGGLPPP and Ser31, Tyr148, Lys197, and Asp198 (Figure 13a). The predicted configuration of IFGGLPPP overlapped significantly with that of LY3502970 (PDB:6XOX) in GLP-1R. A closer look at the effect of IFGGLPPP on GLP-1R revealed that it might increase binding affinity if the derivatized peptide could interact more with TM3, ECL1, and ECL2 (Figure 9a). By continuously adjusting the residue composition and observing the docking results, it is expected to create a better sequence with a higher binding score. The backbone of the IFGGLPPP derivative docked with GLP-1R exhibited a free-curved or complex-curved configuration, including U-shaped (IFGGLPPP), W-shaped (IFPRWPP), S-shaped (IFGGWPPP, IFPGWPP), J-shaped (IFPGWPPP), ç-shaped (IFGGYWPPP), and C-shaped (IFGYGWPPPP) configurations. The multiple interfaces at which these curve configurations interacted with GLP-1R offer the condition of establishing multiple hydrogen bonding and π–π stacking. Among them, the docking results of IFGGWPPP^(N7)^, IFPGWPPP^(N11)^, IFGGYWPPP^(N20)^, and IFGYGWPPPP^(N21)^ were particularly attractive, as discussed below.

### 2.6. The Configuration of IFGGWPPP^(N7)^ and IFPGWPPP^(N11)^ in Docking with GLP-1R

IFGGWPPP^(N7)^ had an S-shaped structure and fit closely with the TM1, TM2, and TM3 axes of GLP-1R. Its “IF” residue interacted with Pro137^TM1^, Leu141^TM1^, and Tyr148^TM1^ to fix the beginning of the peptide, and then the following “GG” residues established hydrogen bonds with Ser31^ECD^ and Tyr205^TM2^. The “WPPP” at the end showed the C-type structure, surrounded by Trp33^ECD^, Thr298^ECL2^, Lys197^TM2^, and Gln234^TM3^. Comparing the predicted configurations of IFGGWPPP and IFGGLPPP, it was found that the π–π interaction between the W residue in IFGGWPPP and Trp33^ECD^ is the key to guiding the entire peptide to form an S-shaped configuration, thereby enhancing the performance of linear tension and the corresponding binding affinity (−10.7 kcal/mol). The layout of IFGGWPPP was similar to LY3502970/V6G (PDB:6XOX), thus its potential to induce GLP-1R activation was expected; however, the terminal of LY3502970 interacted with Tyr220^ECL1^, whereas IFGGWPPP interacted with Gln234^TM3^. When IFGGWPPP^(N7)^ was transformed into IFPGWPPP^(N11)^, it was found that a bend was formed on the proline in the “IFPG” fragment. The linear peptide was thus placed along TM2 to interact with Lys197^TM2^; the terminal “PPP” was further extended to Arg310^TM5^ and Tyr241^TM3^. The overall configuration of IFPGWPPP was like an inverted J shape, and the binding affinity was -11.0 kcal/mol. IFGGWPPP^(N7)^ and IFPGWPPP^(N11)^ were associated with GLP-1R with different curvatures. The S-shape of IFGGWPPP^(N7)^ seemed to be more resilient when the GLP-1R configuration was slightly changed or deformed, but the higher binding affinity of IFPGWPPP^(N11)^ may also have potential benefits. 

### 2.7. The Configuration of IFGGYWPPP^(N20)^ and IFGYGWPPPP^(N21)^ in Docking with GLP-1R

From the related PDB crystal analysis, it was found that both LY3502970 and TT-OAD2 established hydrogen bonds with Tyr220^ECL1^. One end of peptide 5 and PF-06882961 also passed through Trp33^ECD^ and approached ECL1. However, the docking results showed that these plant-derived cyclopeptides and N1~N17 linear peptides did not cross Trp33^ECD^ and Thr298^ECL2^ to interact with residues on ECL1, such as Tyr220 and Gln221. Due to the curvature of the cyclopeptides or linear peptides, in most cases after interacting with Ser31 or Tyr205, the main axis of the backbone then bypassed Trp33^ECD^ and Thr298^ECL2^ to get close to Asn300^ECL2^ instead of crossing Trp33 and Thr298 to extend near the vicinity of ECL1. The residues in the peptide sequence, such as “W, F” have the opportunity to form π–π interactions with Trp33, Glu34, F230, Tyr145, and Tyr148, which may guide the positioning of the peptide. For example, the interaction with F230 led to the configuration of peptides in the direction of Gln234 and Arg310. In order to make the binding affinity of the derived peptide approach −11.9 kcal/mol, establishing an H-bond with Tyr220^ECL1^ may be a way to improve the score. In IFPRWPP^(N13)^, Arg(R), a charged amino acid residue with a longer axis, was introduced into the sequence; however, the results showed that it interacted with Asp198 instead of Tyr220. When the configuration of IFGRGWPPP^(N17)^ was analyzed by the “receptor–ligand interaction” from Discovery Studio (DS) Visualizer, the H-bond between Arg^(N17)^ and Tyr220^ECL1^ was determined. Still, at the same time, Arg^(N17)^ had an unfavorable positive-positive effect on Lys197^(TM2)^. When trying to replace Arg with Tyr(Y) and introduce more Gly(G) and Pro(P) to adjust the curvature of the peptide, it was found that IFGGYWPPP^(N20)^ and IFGYGWPPPP^(N21)^ can establish hydrogen bonds with Tyr220. The binding affinities were −10.9 and −11.7 kcal/mol, respectively. The configuration of IFGGYWPPP was like the French character “Ç”, and it generated H-bonds with Ser31^ECD^, Tyr220^ECL1^, Lys197^TM2^, Thr391^TM7^, and Glu387^TM7^. The configuration of IFGYGWPPPP shown by the docking was a big C shape, and it established hydrogen bonds with Ser31^ECD^, Tyr220^ECL1^, Arg190^TM2^, and Tyr152^TM1^. The saturated curves provided the opportunity to allow IFGGYWPPP^(N20)^ and IFGYGWPPPP^(N21)^ to interact with Tyr220, and therefore established a splendid interaction with GLP-1R. 

Compared with cyclopeptides, linear peptides may display more diverse configurations in GLP-1R and are more flexible in structural design. However, the docking affinity of current attempts still did not surpass that of DdC (−11.9 kcal/mol). Molecules of a specific size may be required to have relevant effects on the multiple helices of GLP-1R and establish sufficient hydrogen bonds. From the data of this series of molecular docking evaluations, peptides composed of 7–10 amino acids were more suitable for developing GLP-1RAs (binding affinity may be better than −10.0 kcal/mol). IFGYGGWPPPP and IFGYGWGPPPP were derived from IFGYGWPPPP^(N21)^, plus an amino acid residue. After docking to GLP-1R, it was found that the peptide consisting of 11 amino acids was transformed into a relatively twisted “G” or “П” shape in order to squeeze into GLP-1R. The binding affinity was no better than that of IFGGYWPPP^(N20)^. 

### 2.8. Summary of the Interaction Mode between IFGGLPPP Derivatives and GLP-1R in Docking

A series of IFGGLPPP-derived peptides exhibited good binding affinity to GLP-1R in docking. The beginning “IF” helped place the peptide between TM1 and TM2, and the “PPP” tail helped stabilize the end of the peptide. Moreover, the number of Gly(G) and Pro(P) can be used to control the rigidity and flexibility of the curve, the Trp(W) and Phe(F) residues can establish a π–π interaction with the receptor, and the Tyr(Y) can form a hydrogen bond in a specific direction. Hydrogen bonding of the PPP tail to Gln234, Arg310, or Arg190 can anchor one end of the linear peptide to GLP-1R, which may also affect downstream reactions of GLP-1R. The docking of this series of peptides with GLP-1R suggests that linear precursors derived from cyclopeptides (e.g., HB) may be another framework that can be involved in GPCR agonist studies in addition to truncated and modified GLP-1 (e.g., peptide 5 of PDB:5NX2) or new nonpeptide molecules. Fragments derived from cyclopeptides appeared to have curvature properties in their structure, corresponding to multiple α-helices of GPCRs in flexible configurations. The synthesis of various IFGGLPPP-derived peptides was relatively easy compared to nonpeptide compounds. This facilitated follow-up research after docking. However, linear peptides may be less stable than cyclopeptides or nonpeptide compounds in physiological settings, and peptides containing residues W and P may be less stable and prone to undergo side reactions. Peptides may be hydrolyzed by enzymes in the gastrointestinal tract and must be tested for tolerance. Some modification strategies targeting N- or C-terminal peptides or specific amino acids may provide ways to avoid peptide hydrolysis or improve bioavailability [53]. Potential peptides after in silico evaluation require more in vitro and in vivo studies, as the binding affinity predicted by docking is insufficient to determine the actual effect on cAMP activation and accumulation. If further experimental data from in vitro and in vivo studies is compared with the docking prediction, it may be possible to find an even better sequence. Based on the framework provided by IFGGLPPP, there is more to be explored. For example, replace I in the original IF with S, T, L, M, V, C, N, or Q, or replace F with W or Y. When the first “Ile” is replaced by other amino acids, the initial positioning of the peptide may not be near Tyr148^TM1^. The conformations of peptides in GLP-1R may exhibit different behaviors. 

### 2.9. Molecular Dynamics Simulation of Potential Cyclic and Linear Peptides on GLP-1R 

**Figure 14:**
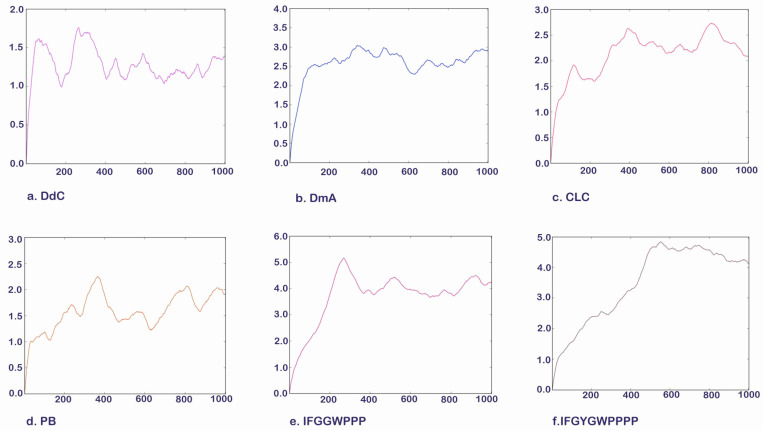
Molecular dynamics simulation. Vertical axis: RMSD (Å). Horizontal axis: number of frames.

**Figure 15:**
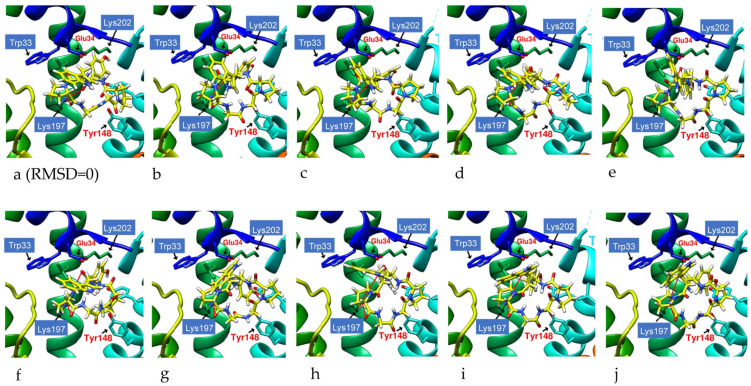
DdC molecular dynamics simulation series with H-bond (red-line) trajectory tracing. Over the course of the dynamic simulation of DdC in GLP-1R, the configuration gradually changes. See images starting from (**a**) (RMSD = 0), through (**b**–**i**) to (**j**). As the RMSD increases, the original hydrogen bonds are weakened by the increase in bond length.

The lowest energy configuration (RMSD = 0) displayed by docking was imported into the UCSF Chimera Molecular Dynamics Simulation, and the structural changes of cyclopeptides and linear peptides in the active region of GLP-1R were further observed over 1000 frames (Figure 14). DdC and PB had lower mean RMSDs relative to DmA and CLC because their configurations were located in a smaller space (Table 4). DmA and CLC had more than 70 atoms whose positions allow for more apparent motion, rotation, and bond twisting; thus, the RMSD is greater than DdC. The average RMSD for cyclic peptides was small, between 1.392 and 2.898 Å. Within this range, the cyclopeptides can maintain the interaction established with GLP-1R at RMSD = 0. This shows that the configuration with the lowest energy (RMSD = 0) obtained by performing ten calculations with AutoDock Vina can be used as a representative of the docking results. Because DdC has conformational changes in small regions, the RMSD plot shows several peaks. During the dynamic simulation of DdC, it was observed that W and Y with longer side chains had a greater effect on the configuration of DdC in GLP-1R (Figure 15). Most of these cyclic peptides can maintain hydrogen bonds with Lys197 and Tyr205 on TM2, but the hydrogen bonds with Gln234 on the outer TM3 may be lost in the process of configuration change. The average RMSD of both IFGGWPPP and IFGYGWPPPP exceeded 4.0 Å because the open N- and C-termini of the linear peptide increased the flexibility of the structure. Linear peptides had a greater range of variation in dynamic observations of 1000 frames. The front end of the sequence can be moved from TM1 to TM2, and the tail end can be moved from TM3 to TM2. However, their general location is still above Lys197. Because GLP-1R is a GPCR, the structure has dynamic variability. The RMSD values of these cyclic peptides and linear peptides can be matched with the changes of GPCRs to produce corresponding interactions. The RMSD value in the range of 1.39–4.23 Å can still allow these cyclic peptides and linear peptides to stay in GLP-1R for some time so as not to immediately exit the active region of GLP-1R due to significant structural changes.

### 2.10. The Potential of D. diandra as a Nutritional Supplement for Blood Sugar and Weight Control

Natural products may benefit people with diabetes, especially when there is no conflict with taking metformin. Drugs acting on the incretin system are prescribed to be used in combination with metformin when needed [5]. It was found by docking that cyclopeptides from *P. heterophylla*, flaxseed, and *D. diandra* have the potential to be GLP-1R agonists, which is relatively difficult to achieve for natural small molecules. If the peptide is required for its medicinal effect, the mixture obtained from alcohol extract, whole powder, leaf protein powder, etc., may be better than boiled herbal tea. Flaxseeds are often consumed as a powder, but *P. heterophylla* and *D. diandra* are mostly cooked as herb soup. Improving how these active ingredients are extracted may increase the efficacy of using natural products. However, natural products may contain trace amounts of toxic saponins, lectins, alkaloids, etc., which require safety verification or pretreatment during the manufacturing process. *P. heterophylla* and flaxseed are used as health foods or dietary supplements in certain regions, but *D. diandra* is mainly used as an herb. *D. diandra* is not as well-known as *P. heterophylla* and flaxseed in terms of daily use. The results of docking with GLP-1R show the research potential of *D. diandra*. The binding affinities of DmA and DdC from *D. diandra* to GLP-1 occupy the first and second positions among the cyclopeptides of these three plants. DdC is a bioactive proline-rich cyclohexapeptide, and a fully synthetic method is currently available. It has been observed to have antimicrobial and anthelmintic properties [54]. It may have more applications than is presently known. In warm and humid regions, *D. diandra* thrives in the field and may have the opportunity to develop into a model plant for peptide production. In particular, the regulation of GLP-1R is related to weight control. Semaglutide is being evaluated as a weight management drug. *D. diandra* may have potential as a dietary supplement for weight management later. In addition to lowering blood sugar, DdC also has great research potential in weight loss research.

### 2.11. Effects of Cyclopeptides on DPP4 and GLP-1R, Two Targets of the Incretin System

From our previous docking analysis, it was found that numbers of cyclopeptides have the potential for DPP4 inhibition, especially HB and PB from *P. heterophylla*, CLA, CLB, and CLC from flaxseed, and DmA and DdC from *D. diandra* with binding affinities of −10.4, −9.6, −9.8, −9.8, −10.0, −10.2, and −10.7 kcal/mol, respectively [45]. The binding affinity of PB, CLB, CLC, DmA, and DdC to GLP-1R is better than DPP4. The binding affinity of DdC to GLP-1R (−11.7 kcal/mol) is more evident than that of DPP4 (−10.7 kcal/mol). Since the catalytic center of DPP4 is located on one side of the large cavity, the cyclopeptide only partially acts on it. On the other hand, cyclopeptides interact with amino acid residues on the seven helical axes of GLP-1R, resulting in more hydrogen bonds, π–π interactions, and a higher binding affinity. According to research, GLP-1R agonists have a higher hypoglycemic effect than DPP4 inhibitors, and it is favorable when the binding affinity of cyclopeptides to GLP-1R is higher than that of DPP4.

We observed that when cyclopeptides are docked with DPP4 and GLP-1R, they can establish different interactions according to the surrounding conditions. The docking result of these plant cyclopeptides with DPP4 and GLP-1R suggested that the regulation of dual targets in the incretin system may be a way or phenomenon for certain natural products to be involved in the hypoglycemic mechanism. We also found that it is possible to design molecules with high binding affinity to DPP4 and GLP-1R, such as IFGGWPPP and IFGWPPP. The molecules of existing DPP4 inhibitor drugs (e.g., vildagliptin, saxagliptin, alogliptin, and linagliptin) are smaller than nonpeptide GLP-1R agonists (LY3502970, TT-OAD2, and PF-06882961). It is more difficult to use small molecules as complete agonists of GLP-1R. The docking results revealed that cyclic and linear peptides with considerable molecular weight and flexibility might have dual effects on DPP4 and GLP-1R. More physiological investigations are needed.

### 2.12. Limitations of the Study

This article discussed the potential of three plant cyclic peptides as GLP-1R agonists in silico. In addition, Table 3 documents the development of linear peptide derivatives of HB as GLP-1R agonists. In Table 3, the interactions of N5-N22 with GLP-1R each included at least two hydrogen bonds (over five on average) and at least one π–π interaction. Such effects are similar to those observed for LY3502970, TT-OAD2, and PF-06882961 that interacted with multiple transmembrane helices as GLP-1R agonists. However, at this stage, the data comes only from molecular modeling, and the information that can be provided is still quite limited. Furthermore, due to the discussion involving 39 compounds, only systematic analysis and observation of the most interesting compounds were carried out. In the future, with the help of more biological experiments, it will be possible to go back to review these docking results or conduct more detailed studies.

## 3. Materials and Methods

Molecular docking can predict the ligand’s binding affinity and corresponding conformation in biological macromolecules. It plays an important role in modern structure-based computer-aided drug design (SB-CADD). AutoDock Vina was used in this study because it uses a sophisticated gradient optimization method to increase calculations’ speed and accuracy and obtain good reproducibility when calculating molecules with multiple rotatable bonds [55]. It is a valuable tool for evaluating the relationship between macromolecular ligand and complex receptors. 

Q Li et al., analyzed the potential of NPC1 derived peptides (e.g., the cyclic peptide Ac-cyclo (CDDFFVYC)-NH2 and linear Ac-DDFFVY-NH2) to inhibit the entry of the Ebola pseudotype virus by targeting the GP protein [56]. V Kounnis et al. demonstrated the cytotoxic activity of microcystin-LR: cyclo (Ala-Leu-MAsp-Arg-Adda-isoGlu-Mdha) from cyanobacteria is due to the interaction with the organic anion-transporting polypeptide (OATP) transporters on the membrane of pancreatic cancer cells [57]. S Pang et al., observed that cyclic-4-Asp cooperates with the hydrophobic pocket in the conical region as an HCV p7 channel blocker [58]. In these studies, AutoDock Vina-assisted assessment of the effect of cyclic peptides on multimeric channels suggests that molecular modeling is a useful tool for calculating the binding affinity and configuration of peptides to GPCRs.

The structure (2D or 3D format) and biological and chemical description of *P. heterophylla*, flaxseed, *D. diandra*, and related GLP-1 agonists were downloaded from PubChem. The three-dimensional crystal structure of GLP-1R (PDB:6X19) was obtained from the RCSB protein data bank as a docking receptor. The downloaded analysis reference included PDB:6X18, 5NX2, 6VCB, 6ORV, 6XOX, 6X1A, and 7PIU. The original 2D files and IFGGLPPP derivatives needed to be further edited or constructed by MarvinSketch, ACD/ChemSketch, ChemDraw, or Avogadro into the 3D format to meet the requirements of the docking software or the form presented by the text. UCSF Chimera 1.13.1 was used because it can compare and match multiple data obtained from PDB, and is a powerful tool for understanding the crystallization data. It also provides structural analysis and editing tools required before molecular docking and a platform to execute the AutoDock Vina_1_1_2 program. In addition, its built-in rendering function can display the docking results with high-resolution 3D images. 

Before docking, the receptor and peptide molecules underwent a “dock prep” procedure, including (1) solvent removal, (2) hydrogen addition, and (3) charge addition under UCSF Chimera. Charges were computed using ANTECHAMBER [59]. The prepared receptor and ligand were then be introduced into the AutoDock Vina. The grid center was set to X = 130.648, Y = 111.191, and Z = 86.086 (the centroid of the original ligand of PDB:6X19). The grid size was set to 40 × 40 × 40. Since the cyclopeptides from the three plants discussed in this paper do not contain disulfide bonds and would not involve the pairing calculation of disulfide bonds, all parameters followed the default settings of AutoDock Vina. The peptide was kept flexible during the docking process, whereas GLP-1R was set as rigid. A set of ten calculations were performed in one docking process, and the configuration with the lowest energy (RMSD = 0) was selected for subsequent analysis as the docking results.

The predicted lowest energy configuration was displayed in GLP-1R, and the hydrogen bonds were marked as a red line by the “Find H-bond” search tool on UCSF Chimera. The search condition for hydrogen bonds was set to “Relax constraints: 0.4 angstroms and 20.0 degrees”. The ligand-containing complexes were presented as colored secondary structures. BIOVIA Discovery Studio (DS) Visualizer’s “show receptor–ligand interactions on a 2D diagram” tool demonstrated the various interactions between the specified peptide and GLP-1R, including hydrogen bonding, π–π stacking, van der Waals force, etc. The pictures from DS Visualizer did not show hydrogen atoms that have no special effect. The hydrogen bonds produced by UCSF Chimera and DS Visualizer may be slightly inconsistent because the search criteria (default settings) were different for the two systems.

The configuration with RMSD = 0 was further analyzed with the Chimera Molecular Dynamics Simulation program (V. Munoz-Roles and J.-D. Marechal designed the interface). The setting conditions of the molecular dynamics simulation were: “Settings = Minimization. Lower RMSD threshold = 1.3. Upper RMSD threshold = 1.8. Steepest descent steps = 100. Steepest descent steps size = 0.02 Å. Conjugate gradient steps = 10. Conjugate gradient step size = 0.02 Å. Start frame = 1. Step size = 4. ending frame = 1001”. The molecular weight, theoretical pI, and hydrophobicity of linear peptides were analyzed with the Peptide Analyzing Tool (Thermo Fisher Scientific). 

## 4. Conclusions 

The GLP-1 receptor is a class B1 GPCR which has received much attention as a target of hypoglycemic drugs. The possibility of a series of plant-derived cyclopeptides from *Pseudostellaria heterophylla*, *Linum usitatissimum*, and *Drymaria diandra* as GLP-1 agonists was explored by docking, and HB (−9.5 kcal/mol), CLC (−10.6 kcal/mol), DmA (−11.2 kcal/mol), and DdC (−11.9 kcal/mol) were found to have good binding affinity. Among them, the performance of DdC:(cyclo)-GGPYWP-(cyclo) from *D. diandra* is the most prominent. Although these are peptides, their molecular weight was much smaller than GLP-1 analogs and was close to the current nonpeptide GLP-1R agonists. Because of the circumferential size of these cyclopeptides, they can enter the transmembrane region of GLP-1R and can interact with amino acids on multiple helix axes to display good performance in docking. In contrast, natural substances with smaller molecular weights may be more difficult to achieve GLP-1R agonism. The cyclopeptides of these three plants are mainly composed of hydrophobic amino acids with no disulfide bonds and only a single ring. In Caryophyllaceae, Annonaceae, etc., many plants have similar cyclopeptides, some of which are also medicinal or edible. These plant cyclopeptides are more readily available than marine cyclic peptides and are not as toxic as some disulfide-containing cyclotides. Generally, natural cyclic peptides are more discussed regarding anticancer, antiviral and antibacterial properties, and less in the context of hypoglycemic or nutritional supplementation. The docking results provide insights into the role of natural cyclopeptides in lowering blood sugar with opportunities as a dietary supplement option for T2D patients. The further study of cyclopeptides and their targets will contribute to their practical application or increase their chances of becoming lead compounds. It can also attract more research on cyclopeptides from Caryophyllaceae, Annonaceae, etc., on GPCRs. In addition, linear peptides obtained by cleavage of cyclopeptides and derivatives created by changing local amino acid residues exhibited a curvilinear configuration on GLP-1R. These linear peptides are flexible and can interact with multiple GLP-1R helical axes. In particular, four linear peptides IFGGWPPP (−10.7 kcal/mol), IFPGWPPP (−11.0 kcal/mol), IFGGYWPPP (−10.9 kcal/mol), and IFGYGWPPPP (−11.7 kcal/mol) derived from the linear peptide precursor of HB (IFGGLPPP) demonstrated their prospects as GLP-1R agonists or backbone candidates. By altering the amino acid sequence, various molecular structures can be rapidly modulated and synthesized for testing, and this may serve as a suggestion for GPCR agonist design. More research is needed.

## Figures and Tables

**Table 1:** The docking result of a series of plant cyclopeptides with GLP-1R.

N	Abbrev.	Molecular Name	Molecular Formula	Binding Affinity	MW
1	HA	Heterophyllin A	(cyclo)-PVIFGIT-(cyclo)	−8.7	727.9
2	HB	Heterophyllin B	(cyclo)-GGLPPPIF-(cyclo)	−9.5	778.9
3	HJ	Heterophyllin J	(cyclo)-AGPVY-(cyclo)	−9.7	487.5
4	PA	Pseudostellarin A	(cyclo)-AGPYL-(cyclo)	−8.1	501.6
5	PB	Pseudostellarin B	(cyclo)-GGGPPFGI-(cyclo)	−10.4	682.8
6	PD	Pseudostellarin D	(cyclo)-GPLILGY-(cyclo)	−9.0	713.9
7	PE	Pseudostellarin E	(cyclo)-GPPLGPVIF-(cyclo)	−9.8	878.1
8	PH	Pseudostellarin H	(cyclo)-GTPTPLFF-(cyclo)	−10.3	861
9	CLA	Cyclolinopeptide A	(cyclo)-ILLPPFFLV-(cyclo)	−9.9	1040.3
10	CLB	Cyclolinopeptide B	(cyclo)-IMLIPPFFV-(cyclo)	−10.3	1058.4
11	CLC	Cyclolinopeptide C	(cyclo)-IM(O)LIPPFFV-(cyclo)	−10.6	1074.4
12	CLF	Cyclolinopeptide F	(cyclo)-LM(O)PFFWVM(O)-(cyclo)	−8.8	1084.4
13	DmA	Drymarin A	(cyclo)-AFPPPFFVI-(cyclo)	−11.2	1016.2
14	DmB	Drymarin B	(cyclo)-GLPFYP-(cyclo)	−9.8	674.8
15	DdA	Diandrine A	(cyclo)-GPWPYF-(cyclo)	−9.5	747.8
16	DdB	Diandrine B	(cyclo)-GPLPLWSS-(cyclo)	−8.8	838
17	DdC	Diandrine C	(cyclo)-GGPYWP-(cyclo)	−11.9	657.7

Binding affinity(kcal/mol), also known as binding energy or docking score, is the most critical data for discussing ligands and receptors in docking. M(O) represents the oxidation state of Met. The molecular weight of the cyclopeptides can be compared with existing nonpeptidic small-molecule GLP-1R agonists. The sequences of these cyclopeptides and their configurations can be used to analyze the origin of the forces between ligands and receptors. MW: molecular weight (g/mol).

**Table 2:** Comparison of the effects of cyclopeptide and nonpeptide GLP-1 agonists on the seven α-helix and adjacent regions of the GLP-1 receptor.

Compound	ECD	TM1	TM2	TM3	TM7	ECL1	ECL2	TM5	TM6
TT-OAD2		Y145, Y148	K197, L201			Y220			
LY3502970	W33	Y148, Y145	Y205		L388	Y220			
PF-06882961	S31	L141	K197	F230 M233	L384 L388	Q221	T298		
HB	W33π	Y148	K197				N300 T298		
CLC	W33π		K197				N300	R310	
DdC	E34π	P137, Y145π	K197						
PB		Y148	R190	Q234					E364π
DmA		Y148π	K197, D198					R310π	

Data for cyclopeptides such as HB are from Figure 8, including hydrogen bonding (amino acids shown) and π interactions (π followed by amino acids).

**Table 3:** Analysis table of docking linear peptide with GLP-1R.

N	Sequence	BA of GLP-1	MW (g/mol)	H-bond, Attractive Charge and Salt Bridge between Linear Peptide and GLP-1R (Data from DS Visualizer)	π–π Interaction
1	PYWP	−9.1	561.644	K197, L141, Y148	-
2	GGPYWP	−9.2	675.748	E34, M233, K197, D198, L141, Y148	-
3	AFPPPFFVI	−9.0	1034.277	N300, Q234, K197, R190	-
4	IFGGLPPP	−8.9	796.973	S31, K197, D198, Y148	-
5	IFGGLPPPP	−9.6	894.091	T298, Q234, S31, Y205, Y148	W33, F230
6	IFGWPPP	−10.3	812.975	Q234, L141	F230
7	IFGGWPPP	−10.7	870.027	Q234, S31, Y205, Y148, K197, P137, L141	T298, W33, Y145
8	IFGGWPFP	−10.2	920.087	Q234, Y148	F230
9	IFGWWPPP	−10.2	999.190	Q234, L141, Y148	F230
10	IFPGWPP	−10.4	812.975	K197, P137, E138	Y145
11	IFPGWPPP	−11.0	910.093	K197, R190, P137, L141, Y241, R310	Y145
12	IFPGWPYP	−10.8	976.153	Y241, K197, R190, P137, L141, Y148	Y145
13	IFPRWPP	−10.4	912.112	Q234, K197, D198, P137, L141, Y148	W33, F230
14	IFPRWPPP	−10.5	1009.230	Y241, K197, R190, P137, L141, Y148	F230, Y145
15	IFPRWPYP	−11.1	1075.290	N300, M233, K197, D198, P137, Y148	F230, Y145
16	IFGRWPPP	−10.0	969.164	W33, D198, Y152	Y145
17	IFGRGWPPP	−10.7	1026.216	Y220, Y241, S31, Y205, Y148, T391	Y145
18	IFPRGWPPP	−11.1	1066.282	Y220, W33, Q234, Y241, D198, R190, Y148, T391	Y145
19	IFGGGWPPPP	−10.3	1024.197	Y205, K197, Y148, E364, T391	F230, Y145
20	IFGGYWPPP	−10.9	1033.205	Y220, Y205, K197, D198, R190, Y148, T391, E387	Y145
21	IFGYGWPPPP	−11.7	1130.323	Y220, S31, K197, D198, R190, Y148, Y152	W33, F230, Y145
22	IFGGYWPPPP	−10.9	1130.323	Y241, K197, R190, Y148, E364, T391	F230, Y145

GGPYWP and AFPPPFFVI are linear peptide forms of DdC and DmA, respectively. IFGGLPPP is the linear precursor of HB. N5-N22 is a series of GLP-1 agonists developed with IFGGLPPP as the backbone. These potential GLP-1 agonists interacted with amino acids on multiple alpha helix axes. The amino acids with which these IFGGLPPP derivatives interacted can be plotted in Figure 3c for comparison. More ideas for designing peptide sequences can be found in these comparisons. Binding affinity (BA), unit: kcal/mol.

**Table 4:** Average RMSD and energy (KJ) obtained from molecular dynamics simulations.

Compound	DdC	DmA	CLC	PB	IFGGWPPP	IFGYGWPPPP
Number of atoms	48	74	76	49	63	82
Average RMSD (Å) over 1000 frames	1.392	2.898	2.083	1.903	4.231	4.121
Average potential energy	736.384	1143.682	995.403	729.980	856.631	1066.311
Average kinetic energy	296.459	557.515	551.055	354.734	412.130	591.659

Data from UCSF Chimera Molecular Dynamics Simulation.

## Data Availability

The data presented in this study are available in article.
